# Supplementary material for: Transdermal Delivery of Macromolecules Using Two-in-One Nanocomposite Device for Skin Electroporation
Source: Pharmaceutics. 2021 Oct 28;13(11):1805. doi: 10.3390/pharmaceutics13111805 (PMC8624044; doi:10.3390/pharmaceutics13111805)
Supplement: Supplementary file 1 [file pharmaceutics-13-01805-s001.zip › pharmaceutics-1421058-supp-for conversion.pdf]

pharmaceutics

Supplementary Materials: Transdermal Delivery of Macromolecules Using Two-in-One Nanocomposite Device for Skin Electroporation

Juliette Simon, Bastien Jouanmiqueou, Marie-Pierre Rols, Emmanuel Flahaut and Muriel Golzio

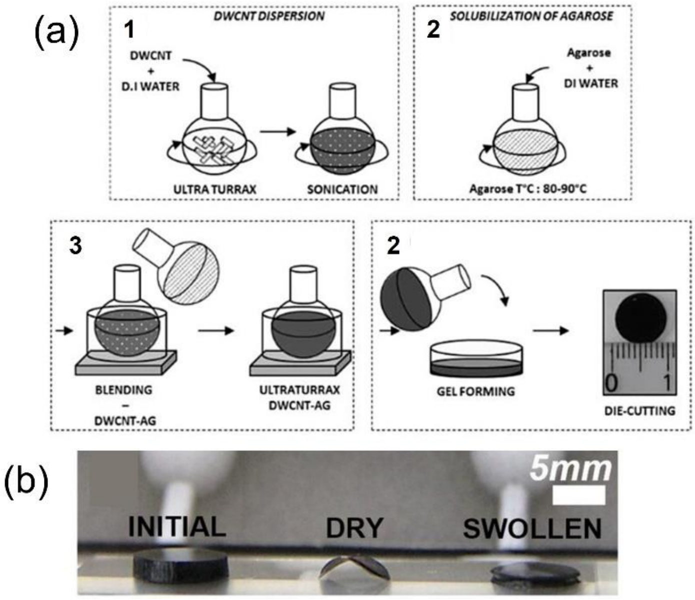

Figure S1. Nanocomposites' preparation. (**a**) Schematic representation of the nanocomposite's synthesis steps. (**b**) Picture of nanocomposites after manufacturing, drying, and swelling in buffer. Adapted from [25].

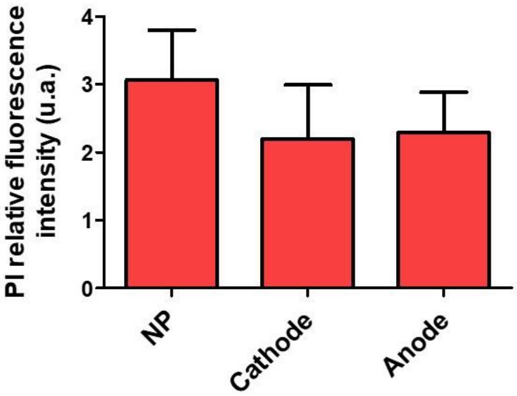

Figure S2. Evaluation of cell death upon EP treatment. One hundred microlitres of PI (100 µM) were applied on the skin 30 min after treatment with a unipolar pulsed electric field to evaluate any deleterious effect of the treatment. The same amount of PI was directly applied on non-pulsed (NP) skins to compare the uptake. PI was left for 30 min at 37 °C before imaging. Quantification of PI uptake in non-pulsed skins, compared to the uptake in treated skins 30 min after treatment. Statistical analysis: one-way ANOVA, Dunnett's post-test comparison vs. NP. Differences are non-significant. Error bars indicate SEM. n = 3.

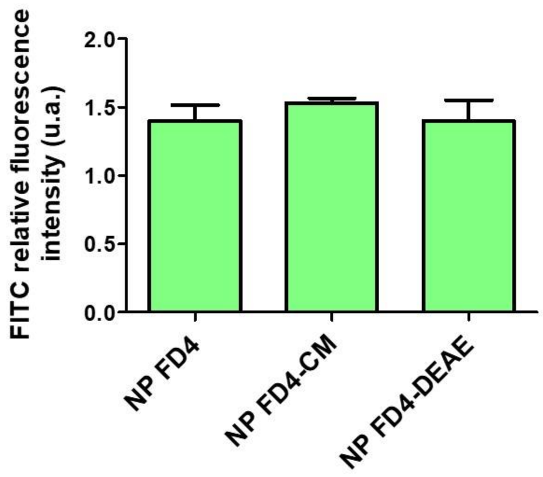

Figure S3. Comparison of the natural uptake of the three FD4 derivates. Platforms loaded with FD4, FD4-CM or FD4-DEAE (1 mM) were applied on non-pulsed skin to quantify the natural uptake of each molecule. Skins were imaged 30 min after. Quantification of FITC uptake in non-pulsed skins when platform loaded with FD4, FD4-CM or FD4-DEAE are applied. Statistical analysis: one-way ANOVA, Turkey's post-test comparison between NP FD4, NP FD4-CM and NP FD4-DEAE. Differences are non-significant. Error bars indicate SEM. n = 3.

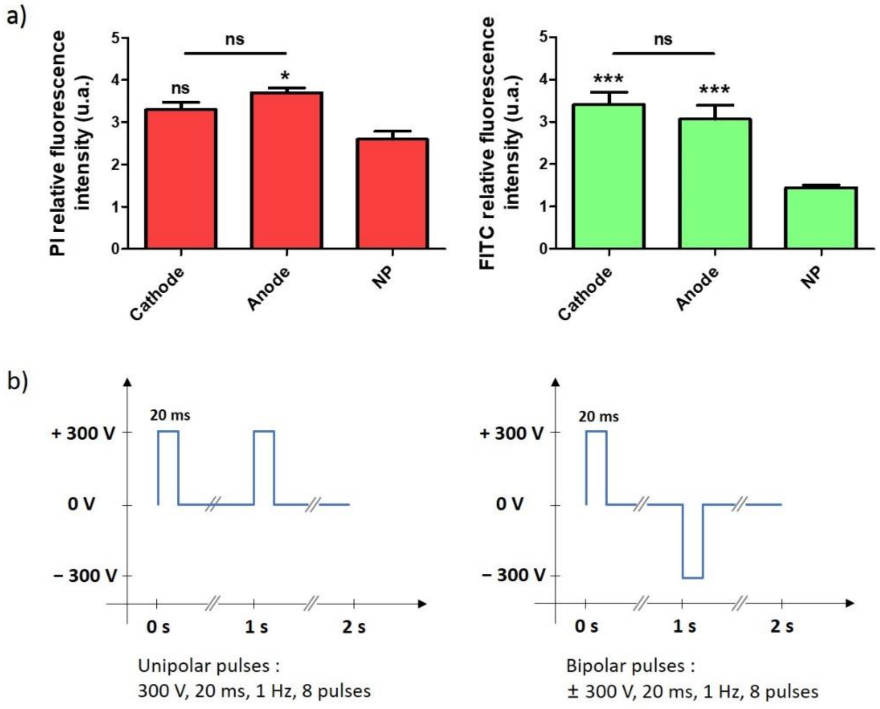

Figure S4. Effect of bipolar pulses on delivery. Platforms loaded with PI (100 μM) and FD4 (1 mM) were used to apply bipolar EP treatment on fresh hairless mice skin explants. Skins were imaged 30 min after. (**a**) Quantification of PI and FITC RFI in skins treated with bipolar pulses when platform loaded with PI and FD4 are applied. Statistical analysis: one-way ANOVA, Turkey's post-test comparison between NP, anode, and cathode side. (Codes signification: * = $p > 0.05$; *** = $p > 0.001$; ns = non-significant). Error bars indicate SEM. n = 3. (**b**) Pulses pattern used for unipolar and bipolar treatment.
